# Supplementary material for: Uncovering associations between pre-existing conditions and COVID-19 Severity: A polygenic risk score approach across three large biobanks
Source: PLoS Genet. 2023 Dec 19;19(12):e1010907. doi: 10.1371/journal.pgen.1010907 (PMC10763941; doi:10.1371/journal.pgen.1010907)
Supplement: S1 Text — (DOCX) [file pgen.1010907.s001.docx]

**S1 Text. Supplementary Methods**

Mendelian Randomization (MR) Analysis

We employed Mendelian Randomization (MR), a statistical method that uses genetic variants as instrumental variables, to infer potential causal relationships between smoking-related traits and COVID-19 susceptibility. Genetic instruments for our exposures—smoking initiation and cigarettes per day—were sourced from genome-wide association study (GWAS) datasets for smoking initiation (https://conservancy.umn.edu/bitstream/handle/11299/201564/SmokingInitiation.txt.gz) and for cigarettes per day (https://conservancy.umn.edu/bitstream/handle/11299/201564/CigarettesPerDay.txt.gz) [1]. We selected variants that reached genome-wide significance and ensured their independence through Linkage Disequilibrium (LD) clumping, utilizing an R^2 threshold of 0.1 within a 250kb window. The outcome data for COVID-19 severity and COVID-19 susceptibility were retrieved from a leave-UKB-out dataset available (COVID-19 Host Genetics Initiative (COVID19-hg GWAS meta-analyses round 7; release date: April 8, 2022; https://storage.googleapis.com/covid19-hg-public/freeze_7/results/20220403/leave_one_out/sumstats/COVID19_HGI_B1_ALL_leave_23andme_and_UKBB_20220403_GRCh37.tsv.gz and https://storage.googleapis.com/covid19-hg-public/freeze_7/results/20220403/leave_one_out/sumstats/COVID19_HGI_B2_ALL_leave_23andme_and_UKBB_20220403_GRCh37.tsv.gz). Our MR analysis encompassed a suite of methods, including MR Egger regression, Weighted Median, Inverse Variance Weighted, Simple Mode, and Weighted Mode [2-5]. Additionally, the Mendelian Randomization Pleiotropy RESidual Sum and Outlier (MR-PRESSO) analysis was conducted to detect and correct for potential horizontal pleiotropy, a pivotal step in identifying any outliers that might influence MR estimates [6].

1. Liu M, Jiang Y, Wedow R, Li Y, Brazel DM, Chen F, et al. Association studies of up to 1.2 million individuals yield new insights into the genetic etiology of tobacco and alcohol use. Nat Genet. 2019;51(2):237-44. Epub 2019/01/16. doi: 10.1038/s41588-018-0307-5. PubMed PMID: 30643251; PubMed Central PMCID: PMCPMC6358542.

2. Burgess S, Butterworth A, Thompson SG. Mendelian randomization analysis with multiple genetic variants using summarized data. Genet Epidemiol. 2013;37(7):658-65. Epub 2013/10/12. doi: 10.1002/gepi.21758. PubMed PMID: 24114802; PubMed Central PMCID: PMCPMC4377079.

3. Bowden J, Davey Smith G, Haycock PC, Burgess S. Consistent Estimation in Mendelian Randomization with Some Invalid Instruments Using a Weighted Median Estimator. Genet Epidemiol. 2016;40(4):304-14. Epub 2016/04/12. doi: 10.1002/gepi.21965. PubMed PMID: 27061298; PubMed Central PMCID: PMCPMC4849733.

4. Hartwig FP, Davey Smith G, Bowden J. Robust inference in summary data Mendelian randomization via the zero modal pleiotropy assumption. Int J Epidemiol. 2017;46(6):1985-98. Epub 2017/10/19. doi: 10.1093/ije/dyx102. PubMed PMID: 29040600; PubMed Central PMCID: PMCPMC5837715.

5. Burgess S, Thompson SG. Interpreting findings from Mendelian randomization using the MR-Egger method. Eur J Epidemiol. 2017;32(5):377-89. Epub 2017/05/21. doi: 10.1007/s10654-017-0255-x. PubMed PMID: 28527048; PubMed Central PMCID: PMCPMC5506233.

6. Verbanck M, Chen CY, Neale B, Do R. Publisher Correction: Detection of widespread horizontal pleiotropy in causal relationships inferred from Mendelian randomization between complex traits and diseases. Nat Genet. 2018;50(8):1196. Epub 2018/07/04. doi: 10.1038/s41588-018-0164-2. PubMed PMID: 29967445.
