# Supplementary material for: Uncovering associations between pre-existing conditions and COVID-19 Severity: A polygenic risk score approach across three large biobanks
Source: PLoS Genet. 2023 Dec 19;19(12):e1010907. doi: 10.1371/journal.pgen.1010907 (PMC10763941; doi:10.1371/journal.pgen.1010907)
Supplement: S1 Fig — (DOCX) [file pgen.1010907.s002.docx]

**S1 Fig**. Scatter plots illustrating PRS and BMI relationships in the MGI cohort (n = 47,257): (A) COVID-19 Severity PRS vs. BMI-PRS; (B) COVID-19 Severity PRS vs. BMI; (C) BMI-PRS vs. BMI. Blue lines represent regression lines, and correlation coefficients (0.065, 0.02, and 0.32, respectively) are displayed in the upper right corner of each panel.
