# Supplementary material for: Uncovering associations between pre-existing conditions and COVID-19 Severity: A polygenic risk score approach across three large biobanks
Source: PLoS Genet. 2023 Dec 19;19(12):e1010907. doi: 10.1371/journal.pgen.1010907 (PMC10763941; doi:10.1371/journal.pgen.1010907)
Supplement: S2 Fig — (DOCX) [file pgen.1010907.s003.docx]

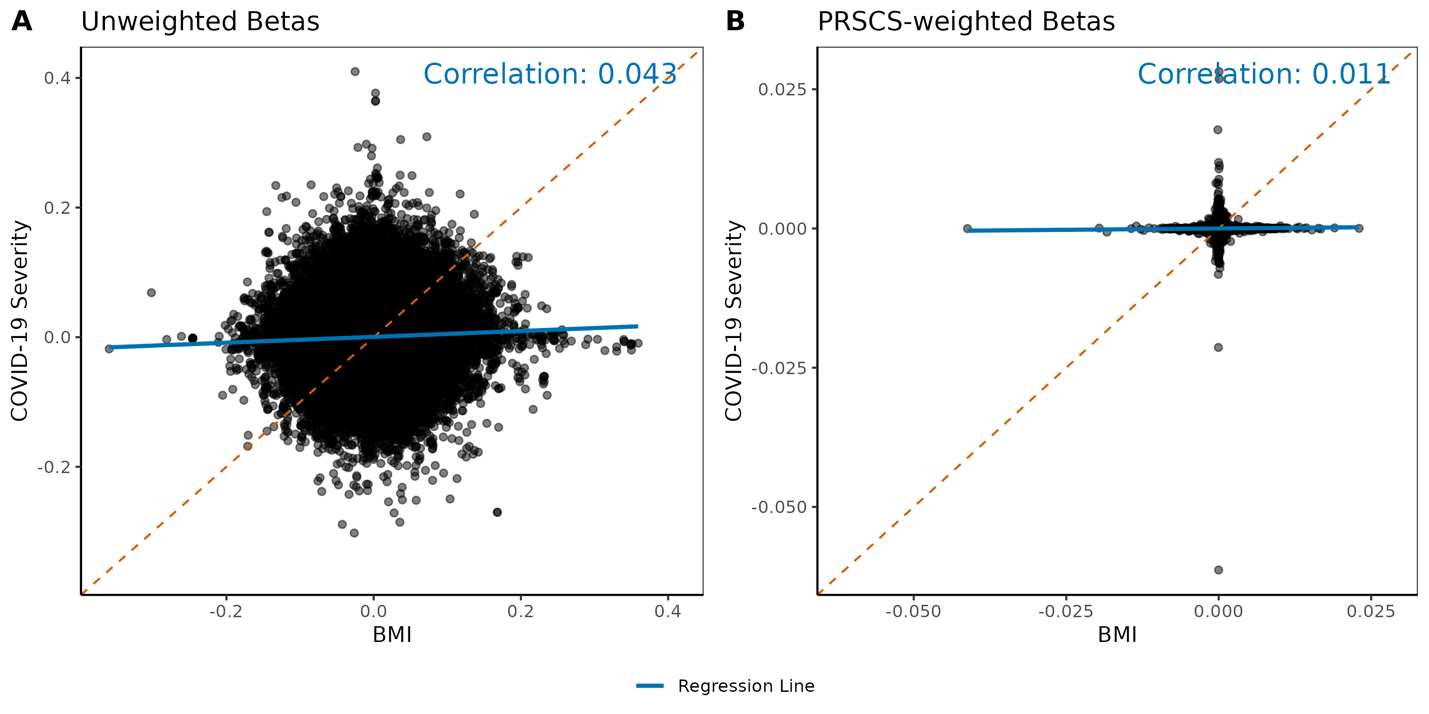


**S2 Fig**. Scatter plot depicting the correlation between BMI and COVID-19 severity GWAS effect sizes (betas) using only overlapping SNPs between BMI and COVID-19 severity GWAS and the PRS-CS LD reference panel (1.1M SNPs). (A) Unweighted betas; (B) PRSCS-weighted betas. Blue lines represent regression lines, while orange lines represent identity lines. Correlation coefficients for unweighted and PRSCS-weighted betas are 0.043 and 0.011, respectively.
