# Supplementary material for: Uncovering associations between pre-existing conditions and COVID-19 Severity: A polygenic risk score approach across three large biobanks
Source: PLoS Genet. 2023 Dec 19;19(12):e1010907. doi: 10.1371/journal.pgen.1010907 (PMC10763941; doi:10.1371/journal.pgen.1010907)
Supplement: S3 Fig — (DOCX) [file pgen.1010907.s004.docx]

**
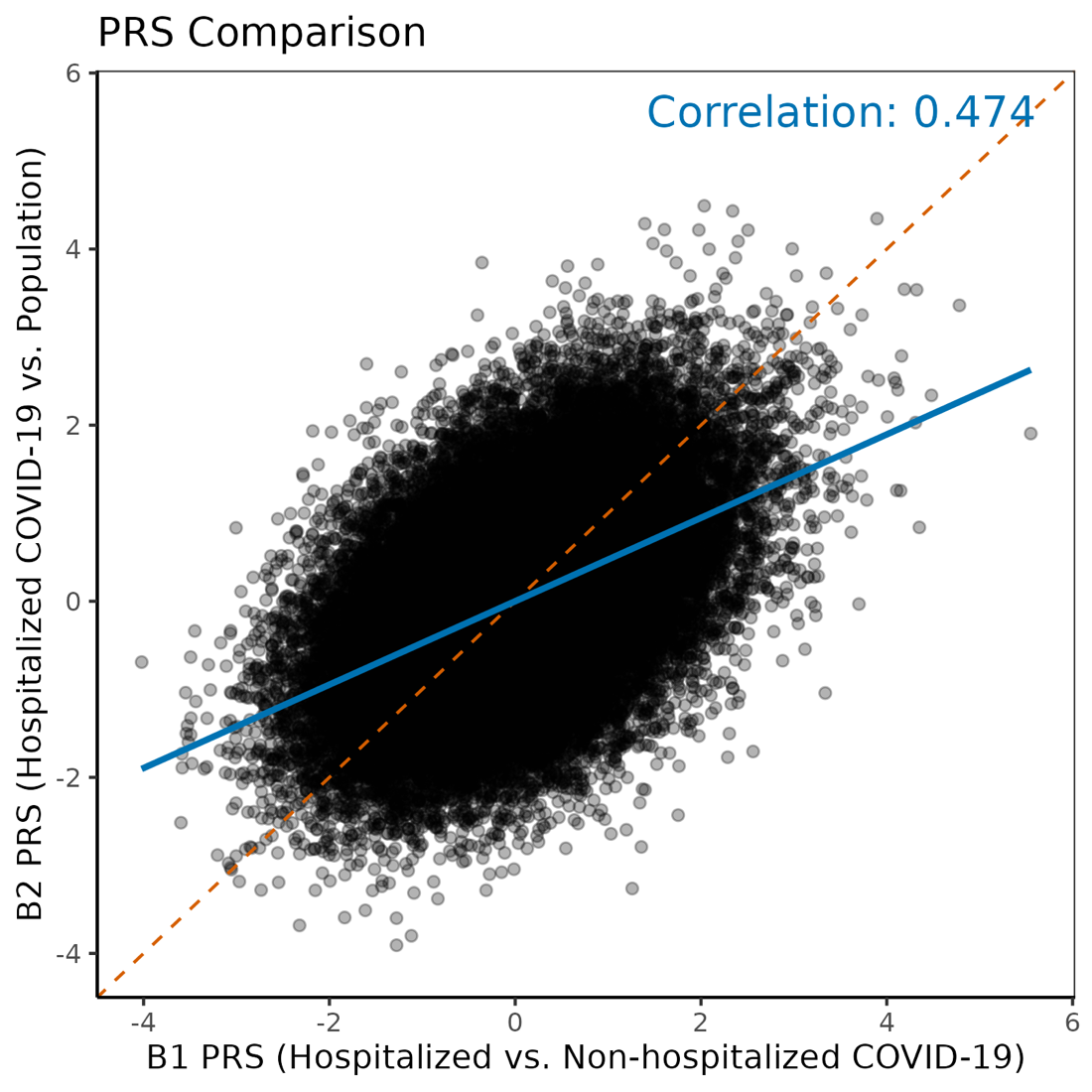
**

**S3 Fig**. Scatter plot depicting the correlation between the “B1_ALL” COVID-19 Severity PRS and the “B2_ALL” COVID-19 Severity PRS in the MGI cohort. Blue lines represent regression lines, while orange lines represent identity lines. Correlation coefficient (0.474) is displayed in the upper right corner.
