# Supplementary material for: Uncovering associations between pre-existing conditions and COVID-19 Severity: A polygenic risk score approach across three large biobanks
Source: PLoS Genet. 2023 Dec 19;19(12):e1010907. doi: 10.1371/journal.pgen.1010907 (PMC10763941; doi:10.1371/journal.pgen.1010907)
Supplement: S5 Fig — (DOCX) [file pgen.1010907.s006.docx]

**S5 Fig. Study specific COVID-19 severity “B2_ALL” PRS PheWAS on pre-pandemic conditions in EUR individuals, adjusted for BMI.** Study-specific PRS PheWAS results for MGI (top, 1694 PheCodes), All of Us (center, 1397 PheCodes), and UK Biobank (bottom, 1388 PheCodes) are shown. PheCodes are only labeled if they have reached nominal significance in one study and phenome-wide significance in another. To avoid overcrowding in the plot, for parent-sibling PheCode combinations, only the top PheCode is labeled. Summary statistics can be found in **S16 Table**. The dashed red line indicates the phenome-wide significance threshold, and the dashed yellow line indicates the nominal significance threshold. The upward/downward orientation of the triangles indicates the positive/negative direction of the estimated association.
