# Supplementary material for: Uncovering associations between pre-existing conditions and COVID-19 Severity: A polygenic risk score approach across three large biobanks
Source: PLoS Genet. 2023 Dec 19;19(12):e1010907. doi: 10.1371/journal.pgen.1010907 (PMC10763941; doi:10.1371/journal.pgen.1010907)
Supplement: S6 Fig — (DOCX) [file pgen.1010907.s007.docx]

**S6 Fig. Meta-analyzed COVID-19 severity “B2_ALL” PRS PheWAS on pre-pandemic conditions in EUR individuals.** A total of 1576 PheCodes that were analyzed in at least two studies are shown without (top) and with adjustment for BMI (bottom). PheCodes are only labeled if they have reached nominal significance in one analysis and phenome-wide significance in another. To avoid overcrowding in the plot, for parent-sibling PheCode combinations, only the top PheCode is labeled. Meta-analysis summary statistics can be found in **S17 and S18 Tables**. The dashed red line indicates the phenome-wide significance threshold, and the dashed yellow line indicates the nominal significance threshold. The upward/downward orientation of the triangles indicates the positive/negative direction of the estimated association.
