# Supplementary material for: Uncovering associations between pre-existing conditions and COVID-19 Severity: A polygenic risk score approach across three large biobanks
Source: PLoS Genet. 2023 Dec 19;19(12):e1010907. doi: 10.1371/journal.pgen.1010907 (PMC10763941; doi:10.1371/journal.pgen.1010907)
Supplement: S7 Fig — (DOCX) [file pgen.1010907.s008.docx]

**S7 Fig.** Forest plots of the BMI-unadjusted association between the PRS for COVID-19 severity and various pre-pandemic phenotypes. Each of the 27 panels (A – AA) represents a phenotype that reached phenome-wide significance in the meta-analysis. Each panel is labeled with its description and phecode. For each phenotype, odds ratios (ORs) and 95% confidence intervals (CIs) are shown for the MGI, UKB, All of Us studies, and the overall meta-analysis. The ORs and 95% CIs are also numerically represented on the right side of each plot. The vertical dashed line represents an OR of 1. The I^2^ statistic, Q statistic, and P-value for heterogeneity are shown for the meta-analysis.

**S7 Fig cont’d**

**S7 Fig cont’d**
