# Supplementary material for: Uncovering associations between pre-existing conditions and COVID-19 Severity: A polygenic risk score approach across three large biobanks
Source: PLoS Genet. 2023 Dec 19;19(12):e1010907. doi: 10.1371/journal.pgen.1010907 (PMC10763941; doi:10.1371/journal.pgen.1010907)
Supplement: S9 Fig — (DOCX) [file pgen.1010907.s010.docx]

| **A ** | **B ** |
| --- | --- |
| **C ** |  |

**S9 Fig. COVID-19 hospitalizations in PRS quartiles of European ancestry individuals of the three cohorts.** Panel A: MGI cohort (n = 47,257); Panel B: UK Biobank cohort (n = 425,787); Panel C: NIH All of Us (n = 47401). Quartiles (1-4) are represented by bars, with the number of hospitalized individuals (numerator) and total individuals (denominator) displayed above. The red line indicates the mean number of hospitalizations across quartiles.
