# Supplementary material for: Uncovering associations between pre-existing conditions and COVID-19 Severity: A polygenic risk score approach across three large biobanks
Source: PLoS Genet. 2023 Dec 19;19(12):e1010907. doi: 10.1371/journal.pgen.1010907 (PMC10763941; doi:10.1371/journal.pgen.1010907)
Supplement: S11 Fig — (DOCX) [file pgen.1010907.s012.docx]

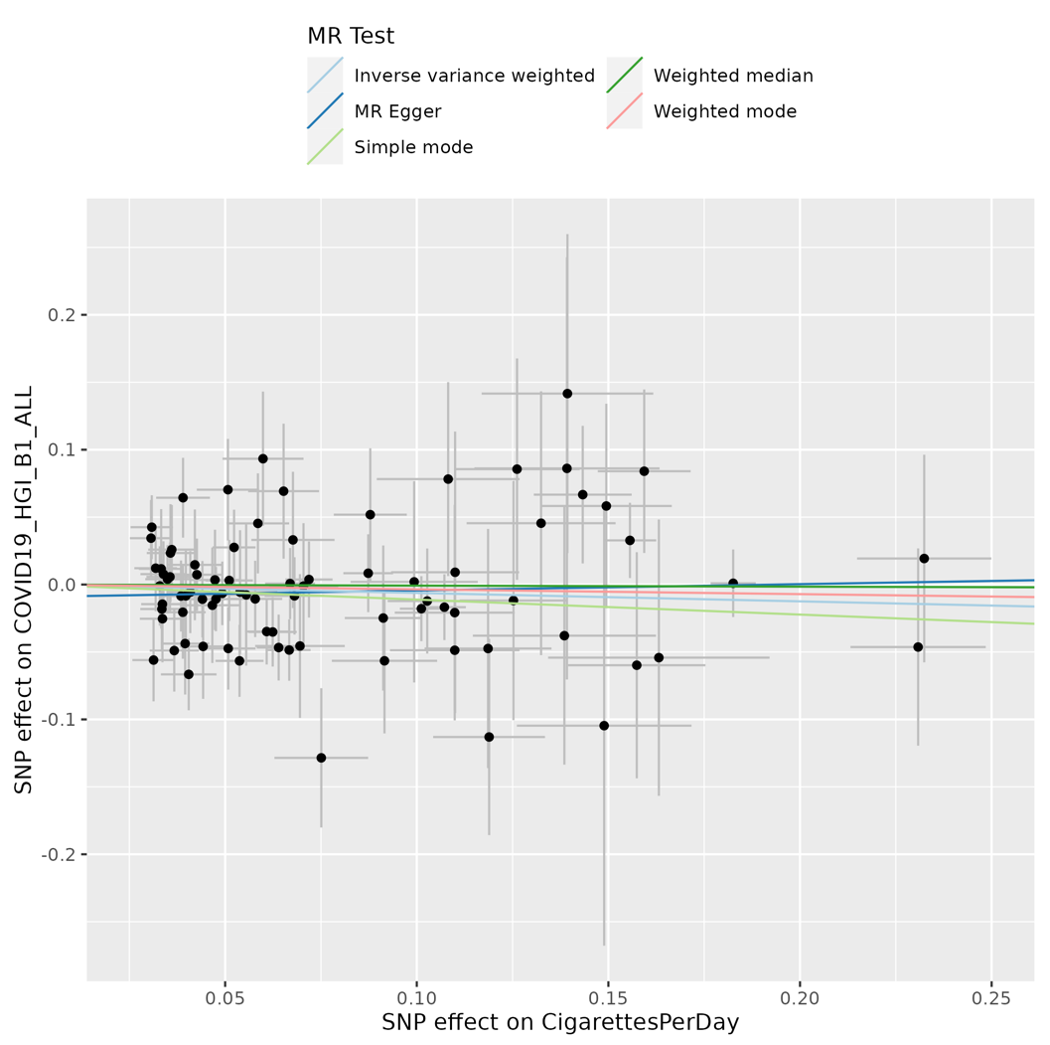


**S11 Fig.** Scatter plot demonstrating the SNP effect of cigarettes smoked per day on COVID-19 severity (B1 ALL). Each dot represents one of 82 SNPs, with the SNP effect on cigarettes smoked per day plotted on the x-axis and the SNP effect on COVID-19 severity depicted on the y-axis. The vertical and horizontal lines encompassing each dot correspond to the confidence intervals for the SNP effects. Mendelian Randomization (MR) estimates from different methods are illustrated as solid lines across the scatter plot: light blue for the Inverse Variance Weighted method, green for the Weighted Median method, blue for the MR Egger method, red for the Weighted Mode method, and light green for the Simple Mode method. The intersection of these lines provides an overall estimate of the causal effect of cigarettes smoked per day on COVID-19 severity.
