# Supplementary material for: Uncovering associations between pre-existing conditions and COVID-19 Severity: A polygenic risk score approach across three large biobanks
Source: PLoS Genet. 2023 Dec 19;19(12):e1010907. doi: 10.1371/journal.pgen.1010907 (PMC10763941; doi:10.1371/journal.pgen.1010907)
Supplement: S12 Fig — (DOCX) [file pgen.1010907.s013.docx]

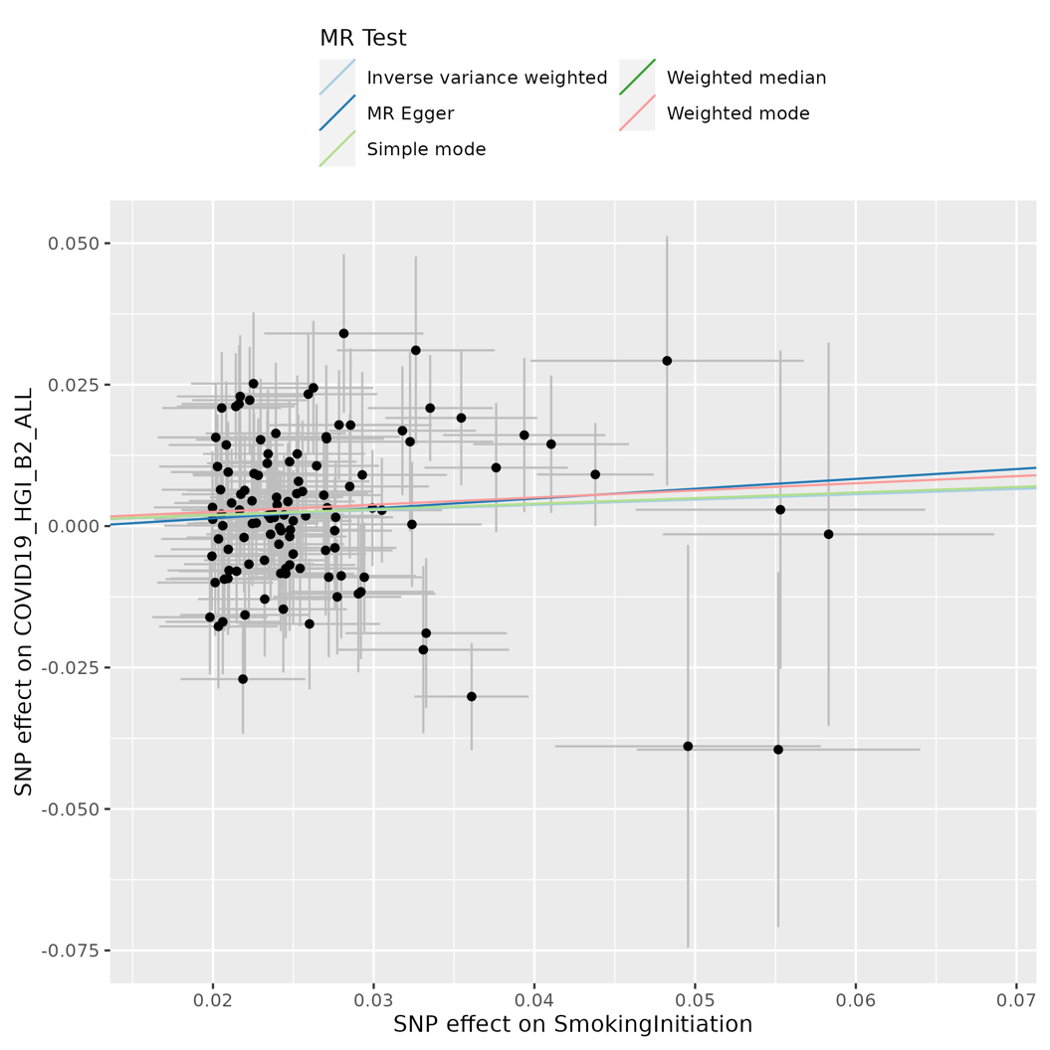


**Fig S12** Scatter plot demonstrating the SNP effect of smoking initiation on COVID-19 susceptibility (B2 ALL). Each dot represents one of 116 SNPs, with the SNP effect on smoking initiation plotted on the x-axis and the SNP effect on COVID-19 severity depicted on the y-axis. The vertical and horizontal lines encompassing each dot correspond to the confidence intervals for the SNP effects. Mendelian Randomization (MR) estimates from different methods are illustrated as solid lines across the scatter plot: light blue for the Inverse Variance Weighted method, green for the Weighted Median method, blue for the MR Egger method, red for the Weighted Mode method, and light green for the Simple Mode method. The intersection of these lines provides an overall estimate of the causal effect of cigarettes smoked per day on COVID-19 severity.
