# Supplementary material for: Uncovering associations between pre-existing conditions and COVID-19 Severity: A polygenic risk score approach across three large biobanks
Source: PLoS Genet. 2023 Dec 19;19(12):e1010907. doi: 10.1371/journal.pgen.1010907 (PMC10763941; doi:10.1371/journal.pgen.1010907)
Supplement: S14 Fig — (DOCX) [file pgen.1010907.s015.docx]

**S14 Fig.** Prevalence of Phenotype Categories in the three analytical datasets. The bar chart displays the pre-pandemic prevalence of 17 phenotype categories among unrelated individuals of European (EUR) ancestry within three biobanks: MGI (green, n = 47,257), UKB (blue, n = 425,787), and All of Us (orange, n = 47,401).
