# Supplementary material for: Uncovering associations between pre-existing conditions and COVID-19 Severity: A polygenic risk score approach across three large biobanks
Source: PLoS Genet. 2023 Dec 19;19(12):e1010907. doi: 10.1371/journal.pgen.1010907 (PMC10763941; doi:10.1371/journal.pgen.1010907)
Supplement: Attachment — Submitted filename: COVID_Severity_PRS_Response2Reviewers_20231111_LF.pdf [file pgen.1010907.s036.pdf]

Response to Reviewer Comments

Title: *Uncovering Associations between Pre-existing Conditions and COVID-19 Severity: A Polygenic Risk Score Approach Across Three Large Biobanks*

Authors: Lars G. Fritsche, Kisung Nam, Jiacong Du, Ritoban Kundu, Maxwell Salvatore, Xu Shi, Seunggeun Lee, Stephen Burgess, Bhramar Mukherjee

Ref: PGENETICS-D-23-00893

We would like to thank the editorial board and the reviewers for their constructive feedback on our resubmission and for giving us an opportunity to address the concerns through a revision. Following are itemized responses to the reviewers' comments. The original comments are in ***italics***, our responses are in **blue,** and the resulting manuscript additions are quoted in **green**. We hope you find the revised manuscript suitable for publication in the PLOS Genetics.

Reviewer #1

The authors used PRS based on COVID GWAS as a proxy of severity of COVID and performed pheWAS in EUR samples from 3 biobanks (UKBB, MGI, and All of US). They found significant association between the PRS and phenotypes related with obesity, metabolic disorders, and cardiovascular conditions and the signal mainly came from UKBB EUR samples. In addition to the analysis performed in EUR samples, the authors also performed pheWAS in non-EUR samples but the signals were either non-significant or negatively correlated with the findings in EUR samples.

While the scholarly pursuit undertaken is undeniably of substantial pertinence, there exist prospects for further enhancement of its methodological robustness.

We thank Reviewer #1 for their kind evaluation of our work. We appreciate the thoughtful comments that we believe have strengthened our revised manuscript.

1.1. First, COVID GWAS contains UKBB EUR samples. The samples that consist of one third of the whole discovery GWAS sample size were then used as the target data in the pheWAS analysis, and most of the signals are from UKBB EUR samples. If the phenotype used in the discovery GWAS and the target data prediction are the same, the overfitting would be very severe for sure. Although the phenotype used in discovery GWAS and pheWAS are different, they still can be correlated and therefore cause overfitting pheWAS results. The authors should rule out the possibility that the signals from the UKBB EUR sample result from overfitting, especially when almost all the significant results are from the UKBB EUR samples. Since the pheWAS in EUR samples is the main findings of this project, all the following analysis and discussion would be questionable if validity of this result cannot be confirmed. One solution could be collaborating with COVID GWAS consortium and getting a GWAS without UKBB samples.

We thank the reviewer for highlighting the importance of avoiding overfitting due to sample overlap. We were conscious of this potential issue from the outset and intentionally selected GWAS summary statistics that **did not** include UK Biobank samples for the PRS analysis of the UK Biobank cohort. To clarify this point, we have added the following sentence to the methods section (pp 10 – 11): "To mitigate the risk of overfitting and to ensure the robustness of our findings, PRSs for the UK Biobank cohort were specifically calculated using GWAS meta-analysis results that excluded UK Biobank samples ('leave_23andme_and_UKBB'): "B1_ALL_leave_23andme_and_UKBB" [12,455 cases vs. 61,144 controls]) and "B2_ALL_leave_23andme_and_UKBB" [40,929 cases vs. 1,924,400 controls]). In contrast, the PRS for the other two cohorts were based on GWAS that included UK Biobank samples."

1.2. Second, I agree with the authors that the inconsistency between EUR and non-EUR results can be caused by the small sample size of non-EUR data and low transferability across populations. I

would like to further point out that for traits that are influenced by many confounding factors, like getting COVID during the pandemic, the PRS transferability across populations can be even lower than traits mainly caused by biological or genetic factors . The COVID GWAS used in this study is mainly based on EUR samples. The PRS based on this GWAS is very likely to be heavily influenced by EUR-specific factors (not only LD structure, but also other confounding factors only existing in the EUR populations) and therefore cannot sufficiently represent the likelihood of getting COVID or the severity of COVID in non-EUR populations. It could be another reason for signals from the AFR and EUR samples being negatively correlated.

We appreciate the reviewer's insightful comments highlighting the potential limitations of PRS applicability across different populations. Considering this, we have amended the Discussion section (pp 25 – 26) to address these concerns more directly:

"Secondly, we did not assess the predictive performance of the COVID-19 severity PRS as it is usually recommended for newly developed PRS [67] due to a lack of well-characterized COVID-19 cases/severity and small sample sizes. Instead, we relied only on the discovery GWAS and the applied PRS method, i.e., any biases or confounding in the underlying GWAS may have also biased the resulting PRS. In particular, the predictive accuracy of PRS is likely diminished for non-European individuals due to the GWAS being based primarily on European samples, where EUR-specific environmental and socio-economic factors, in addition to genetic factors, may significantly influence COVID-19 severity. Thirdly, our approach did not work for non-European subsets, which could be due to their substantially smaller sample sizes and the well-established lack of transportability of PRS across diverse populations [68]. This underscores the need to establish larger, more diverse populations to expand the investigation of a COVID-19 severity PRS to a broader group of individuals. Finally, we did not account for selection bias in the three cohorts, which could explain some of the heterogeneity we observed in the meta-analysis. For example, MGI is a hospital-based cohort enriched for patients undergoing surgery [46], and UKB is a population-based cohort that was reported to have a "healthy volunteer" selection bias [69]. At the same time, All of Us has purposefully oversampled certain underrepresented subgroups [44,70]. While many of our PheWAS results align with previous reports, moving forward, it is imperative to include and analyze more representative samples of non-European populations and to apply ancestry aware PRS methods to improve the accuracy and applicability of PRS PheWAS in diverse ancestry groups."

1.3. I also agree with the authors that the correlation between the PRS and the actual severity of COVID could not be tested with the current data due to the existing poor phenotyping of COVID. Therefore, it would be great if the author could have other proxies of severity of COVID to support the findings based on the COVID PRS.

We acknowledge the reviewer's point regarding the limitation imposed by poor phenotyping of COVID-19 severity in our dataset. As suggested, we agree that the inclusion of alternative severity proxies could enrich the analysis. While our current dataset restricts us to hospitalization status, we advocate for future studies to encompass a more nuanced array of clinical endpoints, such as mechanical ventilation, ICU admission rates, and the presence of specific immune biomarkers. This recommendation has been added to the discussion section to guide subsequent research (p 25):

"Future research should strive for a more standardized definition of COVID-19 severity, incorporating additional proxies such as mechanical ventilation requirements, ICU admissions, or specific immune biomarkers, to improve the evaluation of severity PRS models and facilitate cross-study comparisons."

In conclusion, while the authors' work addressed an important topic of the relationships between COVID severity PRS and various phenotypic traits, the study would greatly benefit from addressing the aforementioned concerns to fortify the overall robustness and reliability of its findings.

We thank the reviewer for their guidance and have addressed the points raised to enhance the integrity and clarity of our work.

Reviewer #2

Report: Uncovering Associations between Pre-existing Conditions and COVID-19 Severity: A Polygenic Risk Score Approach Across Three Large Biobanks

The main contribution of this paper is, Authors investigated the use of polygenic risk scores (PRS) as reliable proxies of COVID-19 severity across three large biobanks: the Michigan Genomics Initiative (MGI), UK Biobank (UKB), and NIH All of Us, to identify associations between pre-existing conditions and COVID-19 severity. By utilizing PRS as a proxy for COVID-19 severity, Authors identified known risk factors and novel associations between pre-existing clinical phenotypes and COVID-19 severity.

We thank Reviewer #2 for their insightful feedback on our manuscript. Their suggestions have been instrumental in refining our study.

2.1. Authors performed the analysis stratified by Biobanks due to varying sampling strategies in these Biobanks. It would be great to include the details in the paper such as how they are not comparable with each other.

We agree that it's important to clarify that the three biobanks we've analyzed recruit their participants in very different ways. The MGI is based in a hospital setting and tends to include individuals with specific health issues [PMID: 36819667]. The UK Biobank's participants, despite being randomly selected from the population, are generally healthier than the average person in the UK. This discrepancy is known and could lead to a lower rate of reported health issues [PMID: 28641372]. The All of Us Research Program uses a mix of open invitations and partnerships with healthcare provider organizations to ensure a diverse and representative set of participants [PMID: 31412182].

We added the following to the Discussion section (p 22):

"In examining the distribution of diagnoses across various categories of diseases in unrelated European ancestry cohorts from hospital-based (MGI), population-based (UKB), and the All of Us cohorts, certain patterns emerge, as illustrated in **Fig S14**. Generally, the MGI cohort exhibits a higher proportion of affected individuals across all categories, reflective of its hospital-based nature [PMID: 36819667]. In contrast, the UKB data, representing a population-based sample, consistently reports lower diagnosis rates, especially for congenital anomalies [PMID: 28641372]. The All of Us cohort demonstrates intermediate values reflective of their recruitment, a mix of open invitations and partnerships with healthcare provider organizations [PMID: 31412182]. These observations highlight the variability in health condition prevalence across different cohort types and underscore the importance of considering the cohort source and recruitment strategies when interpreting disease frequency data."

2.2. Would it be possible to describe the phenotypic categories in each bank separately with the help of a plot?

The reviewer raised an important point we have addressed by creating a new bar plot that compares the prevalence of phenotype categories across the three cohorts. This visualization can be found in **Fig R2.1** (**Fig S14** in the manuscript). It clearly shows the differences in disease prevalence discussed in 2.1 and visually supports the sentiment of the impact of recruitment strategies on the prevalence of health conditions.

We added this new plot and referred to it in the new discussion section (see 2.1):

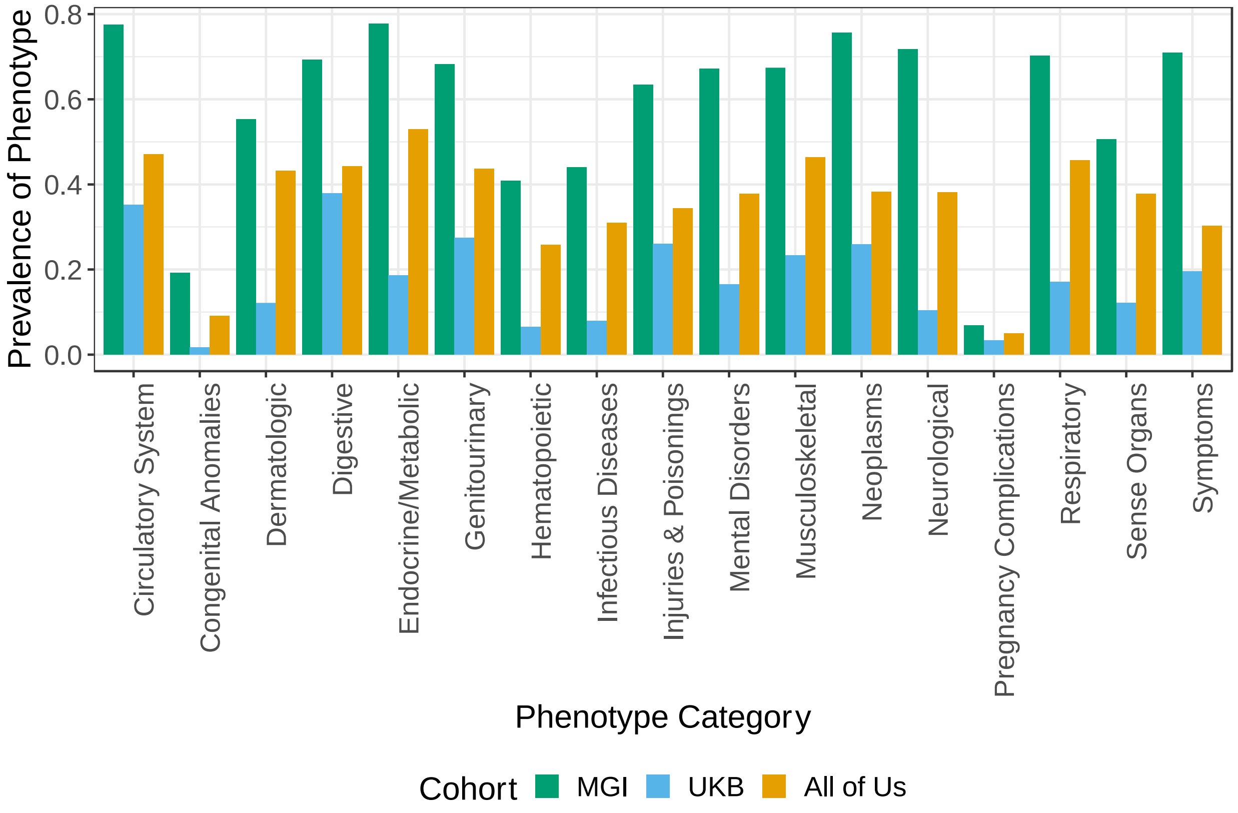

Fig R2.1: Prevalence of Phenotype Categories in the three analytical datasets. The bar chart displays the pre-pandemic prevalence of 17 phenotype categories among unrelated individuals of European (EUR) ancestry within three biobanks: MGI (green, n = 47,257), UKB (blue, n = 425,787), and All of Us (orange, n = 47,401).

2.3. In addition to the table, it would be great to show the association of phenotypes, PheWAS results with the help of plot such as Forest plot.

Thank you for the suggestion. To complement the supplementary tables, we added forest plots of the significant associations of the meta-analysis to the supplementary material (**Fig R2.2 and R2.3, Fig S7 and S8)**.

Fig R2.2: Forest plots of the BMI-unadjusted association between the PRS for COVID-19 severity and various pre-pandemic phenotypes. Each of the 27 panels (A – AA) represents a phenotype that reached phenome-wide significance in the meta-analysis. Each panel is labeled with its description and phecode. For each phenotype, odds ratios (ORs) and 95% confidence intervals (CIs) are shown for the MGI, UKB, All of Us studies, and the overall meta-analysis. The ORs and 95% CIs are also numerically represented on the right side of each plot. The vertical dashed line represents an OR of 1. The I^2 statistic, Q statistic, and P-value for heterogeneity are shown for the meta-analysis.

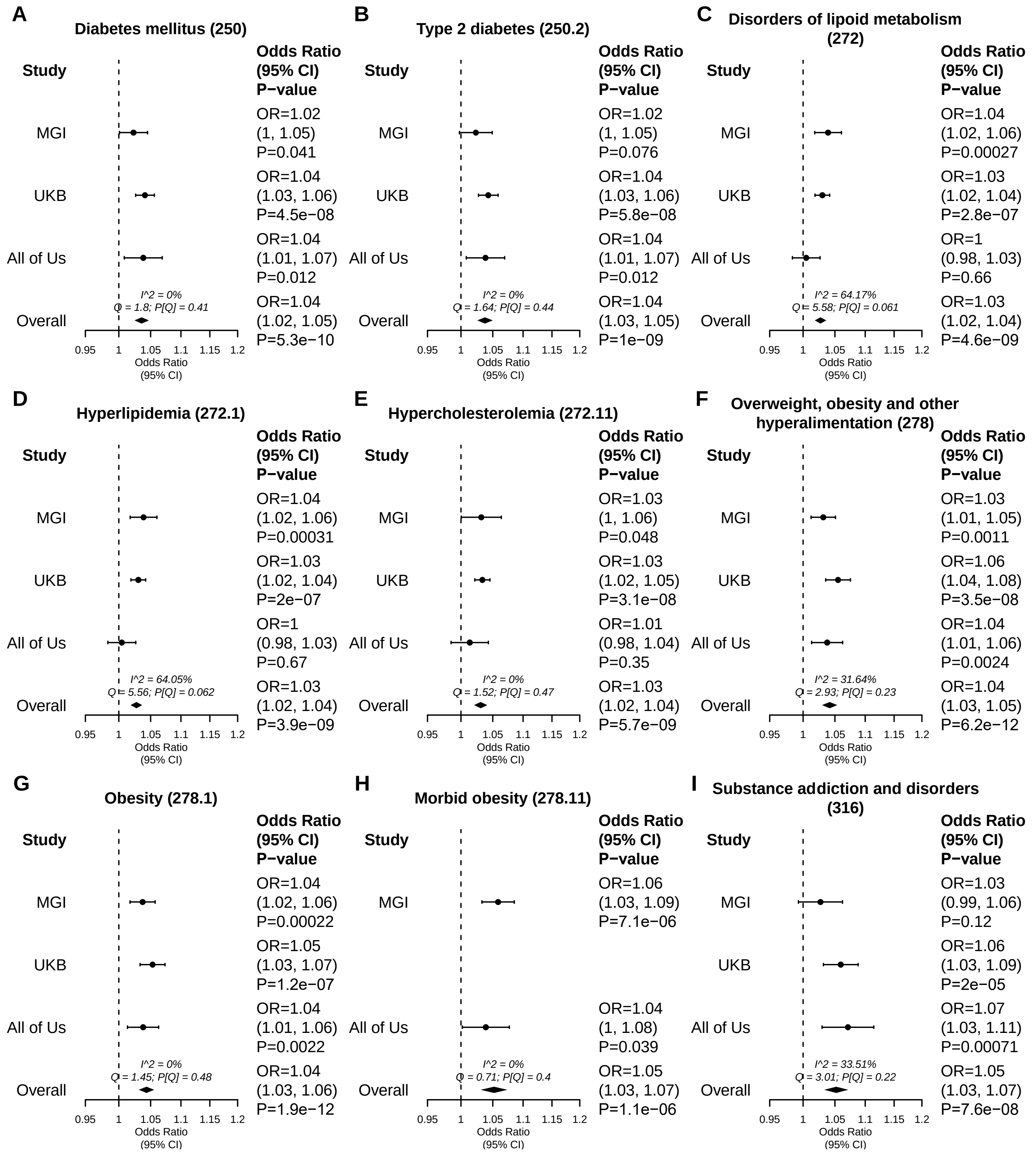

Fig R2.2 cont'd

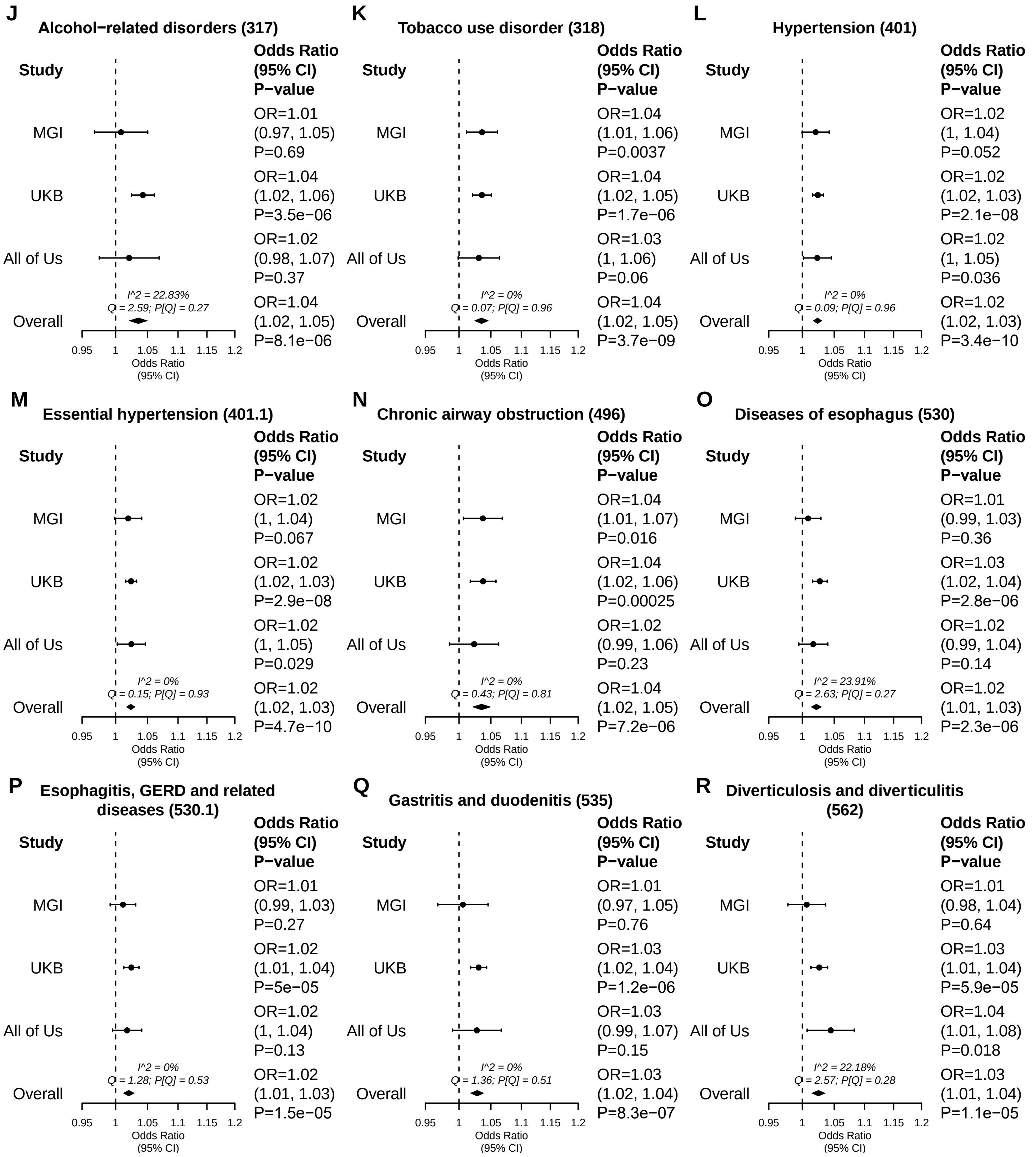

Fig R2.2 cont'd

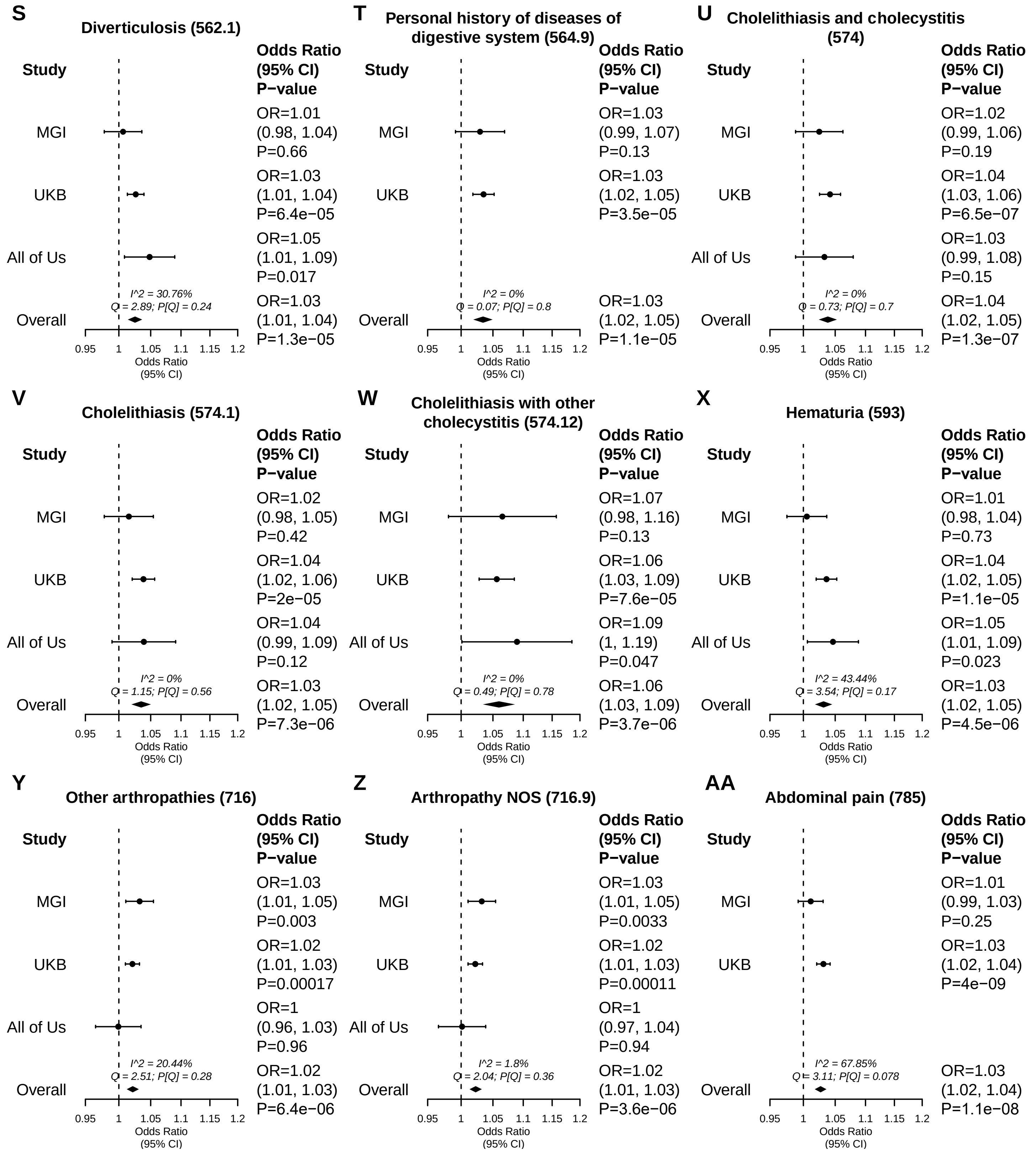

Fig R2.3: Forest plots of the BMI-adjusted association between the PRS for COVID-19 severity and various pre-pandemic phenotypes. Each of the 10 panels (A – J) represents a phenotype that reached phenome-wide significance in the meta-analysis adjusted for BMI. Each panel is labeled with its description and phecode. For each phenotype, odds ratios (ORs) and 95% confidence intervals (CIs) are shown for the MGI, UKB, All of Us studies, and the overall meta-analysis. The ORs and 95% CIs are also numerically represented on the right side of each plot. The vertical dashed line represents an OR of 1. The I^2 statistic, Q statistic, and P-value for heterogeneity are shown for the meta-analysis.

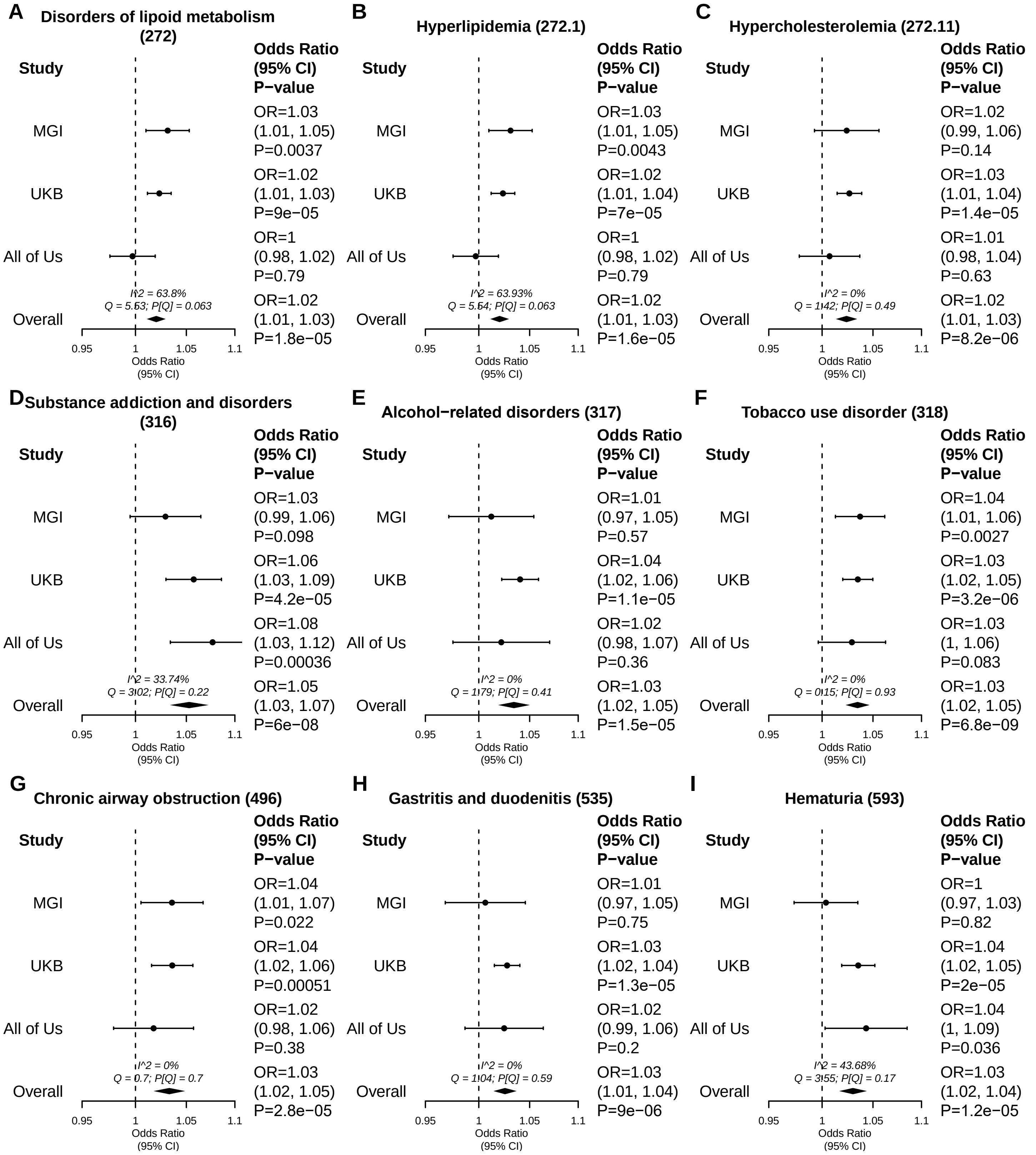

Fig R2.3 cont'd

J

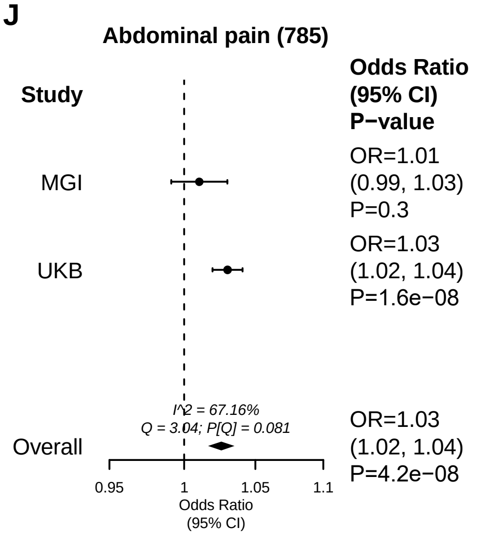

2.4. Please include the details of the summary statistics on COVID-19 severity such the details of the participants, summary of the analysis etc.

We now added details to our method section to include information on participants and provided a concise summary of the underlying analysis for COVID-19 severity and susceptibility GWAS meta-analysis (pp 10 – 11):

"We downloaded the GWAS meta-analysis summary statistics on COVID-19 severity from the COVID-19 Host Genetics Initiative (COVID19-hg GWAS meta-analyses round 7; release date: April 8, 2022; also see **Web Resources**). We considered summary statistics from two GWAS meta-analyses: (1) "B1_ALL": hospitalized COVID-19 versus not hospitalized COVID-19 ("B1_ALL_leave_23andme" [16512 cases vs. 71321 controls] and (2) "B2_ALL": hospitalized COVID-19 versus population controls ("B2_ALL_leave_23andme" [44,986 cases vs. 2,356,386 controls]. To mitigate the risk of overfitting and to ensure the robustness of our findings, PRSs for the UK Biobank cohort were specifically calculated using GWAS meta-analysis results that excluded UK Biobank samples ('leave_23andme_and_UKBB'): "B1_ALL_leave_23andme_and_UKBB" [12,455 cases vs. 61,144 controls]) and "B2_ALL_leave_23andme_and_UKBB" [40,929 cases vs. 1,924,400 controls]). In contrast, the PRS for the other two cohorts were based on GWAS that included UK Biobank samples. The underlying meta-analyses utilized a standard association model, including covariates for age, sex, the first 20 principal components (PCs), and study-specific technical covariates, excluding heritable risk factors and comorbidities. Each contributing cohort conducted GWAS under this framework, employing the SAIGE software [PMID: 30104761] to account for relatedness and case-control imbalance. For a comprehensive account of the participant demographics and individual study contributions, see **Table S1** in the supplementary material, which lists sample sizes and ancestry data for the "B1_ALL" meta-analysis."

2.5. Even though PRS-CS is a well-known PRS development technique, it would be good to include short summary of the algorithm in the paper.

We added a summary of the PRS-CS algorithm to the methods section (p 11):

"We used the software package "PRS-CS" [37] to define PRS weights based on a Bayesian regression framework employing continuous shrinkage (CS) priors. Briefly, PRS-CS adjusts the SNPs 'effect sizes to account for their associations with the trait of interest and the local LD patterns,

thereby resulting in a PRS that more accurately reflects the complex genetic architecture of a trait. It does not require individual-level data but integrates GWAS summary statistics with a provided, precomputed, ancestry-specific LD reference panel for up to 1,117,425 common, non-ambiguous, autosomal SNPs based on samples of the UK Biobank (see **Web Resources**). We opted for PRS-CS because it has demonstrated superior performance to other PRS methods, likely attributable to its adaptable modeling assumptions [55].”

2.6. Please include in the discussion section how this paper is different from similar papers in the literature such as if we use only the PRS with known loci.

We have expanded our discussion to highlight the distinctiveness of our research within the literature (pp 24 – 25):

“Our study's application of a comprehensive GWAS-derived PRS for PheWAS is novel in COVID-19, mirroring successful strategies in other genomic research areas [PMID: 32392212, 29779563, 37663543]. Unlike most studies where COVID-19 risk SNPs have been used as weak instruments [PMID: 34655949], in our research, a PRS serves as a robust proxy for the severity of COVID-19. This is particularly significant given the availability of accurate PRS data for a large number of individuals, contrasting with the often poor quality or incompleteness of COVID-19 outcome data. By broadly capturing genetic variations related to COVID-19 outcomes, our PRS expands risk prediction capabilities beyond what is possible with analyses restricted to known loci like *ACE2* and *TMPRSS2*. This agnostic approach aligns with the aims of initiatives such as the COVID-19 Host Genetics Initiative, which seeks to discover genetic factors impacting patient outcomes [PMID: 32393819], underscoring the value of wide-ranging genetic investigations in understanding disease risks and informing clinical decisions.”

Reviewer #3

The manuscript explores the role of genetic factors in determining the severity of COVID-19 outcomes, utilizing Polygenic Risk Scores (PRS) to predict COVID-19 severity. The authors analyzed genetic data from over half a million individuals across three large biobanks, aiming to identify individuals at high risk of severe illness due to COVID-19. By leveraging PRS, the study seeks to overcome challenges posed by data availability and quality, aiming to inform targeted interventions and prevention measures. The approach could potentially facilitate personalized healthcare, but I have few concerns:

We thank Reviewer #3 for their expert feedback, which has been invaluable in elevating the clarity and rigor of our revised manuscript.

3.1. Causal Inference: The authors aim to shed light on the shared genetic susceptibility of COVID-19 severity and pre-existing conditions. However, establishing associations through PRS does not necessarily imply causality. The study would benefit from a Mendelian Randomization (MR) approach to infer causality and better inform intervention strategies.

We appreciate the suggestion to employ a Mendelian Randomization (MR) approach to infer causality. In alignment with this recommendation, we have conducted supplementary MR analyses to explore the potential causal relationship between smoking behaviors and COVID-19 outcomes. Utilizing various statistical methods (MR Egger, Weighted Median, Inverse Variance Weighted, Simple Mode, Weighted Mode, and MR-PRESSO) and a robust set of instrumental variables (117/116 SNPs for smoking initiation, 82/83 SNPs for the number of cigarettes smoked per day), we have systematically assessed causation. The results indicate that there is no significant causal relationship between genetic predisposition to smoking initiation and the severity of COVID-19 outcomes. Additionally, while the MR analyses provided a marginal indication of a genetic link between cigarette consumption and COVID-19 susceptibility, the evidence is not consistently strong across different MR methods. These findings have been integrated into the revised manuscript to clarify the limitations of causal interpretations derived from our original PRS findings.

We added the follow-up analysis to the Discussion section (pp 21 – 22):

"To follow up on our findings, we performed Mendelian Randomization (MR) analyses and applied a range of statistical methods, including MR Egger, Weighted Median, Inverse Variance Weighted (IVW), Simple Mode, and Weighted Mode, to assess the genetic evidence for a causal relationship between smoking-related traits and COVID-19 outcomes (**Methods R3, Supplementary Methods**). For both smoking initiation and cigarettes per day, the majority of MR analyses did not demonstrate significant causation with COVID-19 severity (B1) or susceptibility (B2), with p-values generally exceeding the nominal significance threshold, indicating no robust genetic causal effect. For the B2 outcome, the IVW method yielded a marginally significant p-value (p=0.023) for the number of cigarettes smoked per day, a finding corroborated by the MR-PRESSO (p=0.025 pre-outlier correction; p=0.029 post-outlier correction; **Fig R3.1 – R3.4 [Fig S10 - 14], Tables R3.1 – R3.4, [Table S20]**). The observed effect sizes were relatively small, suggesting only weak evidence of causality between cigarette consumption and increased COVID-19 susceptibility."

The corresponding methods, tables and figures were added to the supplementary material as **Supplementary Methods, Table S20 and Fig S10 – 14**.

Methods R3 - Mendelian Randomization (MR) Analysis

We employed Mendelian Randomization (MR), a statistical method that uses genetic variants as instrumental variables, to infer potential causal relationships between smoking-related traits and COVID-19 susceptibility. Genetic instruments for our exposures—smoking initiation and cigarettes per day—were sourced from genome-wide association study (GWAS) datasets for smoking initiation (https://conservancy.umn.edu/bitstream/handle/11299/201564/SmokingInitiation.txt.gz) and for cigarettes per day (https://conservancy.umn.edu/bitstream/handle/11299/201564/CigarettesPerDay.txt.gz) [PMID: 30643251]. We selected variants that reached genome-wide significance and ensured their independence through Linkage Disequilibrium (LD) clumping, utilizing an R^2 threshold of 0.1 within a 250kb window. The outcome data for COVID-19 severity and COVID-19 susceptibility were retrieved from a leave-UKB-out dataset available (COVID-19 Host Genetics Initiative (COVID19-hg GWAS meta-analyses round 7; release date: April 8, 2022; https://storage.googleapis.com/covid19-hg-public/freeze_7/results/20220403/leave_one_out/sumstats/COVID19_HGI_B1_ALL_leave_23andme_and_UKBB_20220403_GRCh37.tsv.gz and https://storage.googleapis.com/covid19-hg-public/freeze_7/results/20220403/leave_one_out/sumstats/COVID19_HGI_B2_ALL_leave_23andme_and_UKBB_20220403_GRCh37.tsv.gz). Our MR analysis encompassed a suite of methods, including MR Egger regression, Weighted Median, Inverse Variance Weighted, Simple Mode, and Weighted Mode [PMID: 24114802, 27061298, 29040600, 28527048]. Additionally, the Mendelian Randomization Pleiotropy RESidual Sum and Outlier (MR-PRESSO) analysis was conducted to detect and correct for potential horizontal pleiotropy, a pivotal step in identifying any outliers that might influence MR estimates [PMID: 29967445].

Fig R3.1 Scatter plot demonstrating the SNP effect of smoking initiation on COVID-19 severity (B1 ALL). Each dot represents one of 117 SNPs, with the SNP effect on smoking initiation plotted on the x-axis and the SNP effect on COVID-19 severity depicted on the y-axis. The vertical and horizontal lines encompassing each dot correspond to the confidence intervals for the SNP effects. Mendelian Randomization (MR) estimates from different methods are illustrated as solid lines across the scatter plot: light blue for the Inverse Variance Weighted method, green for the Weighted Median method, blue for the MR Egger method, red for the Weighted Mode method, and light green for the Simple Mode method. The intersection of these lines provides an overall estimate of the causal effect of cigarettes smoked per day on COVID-19 severity.

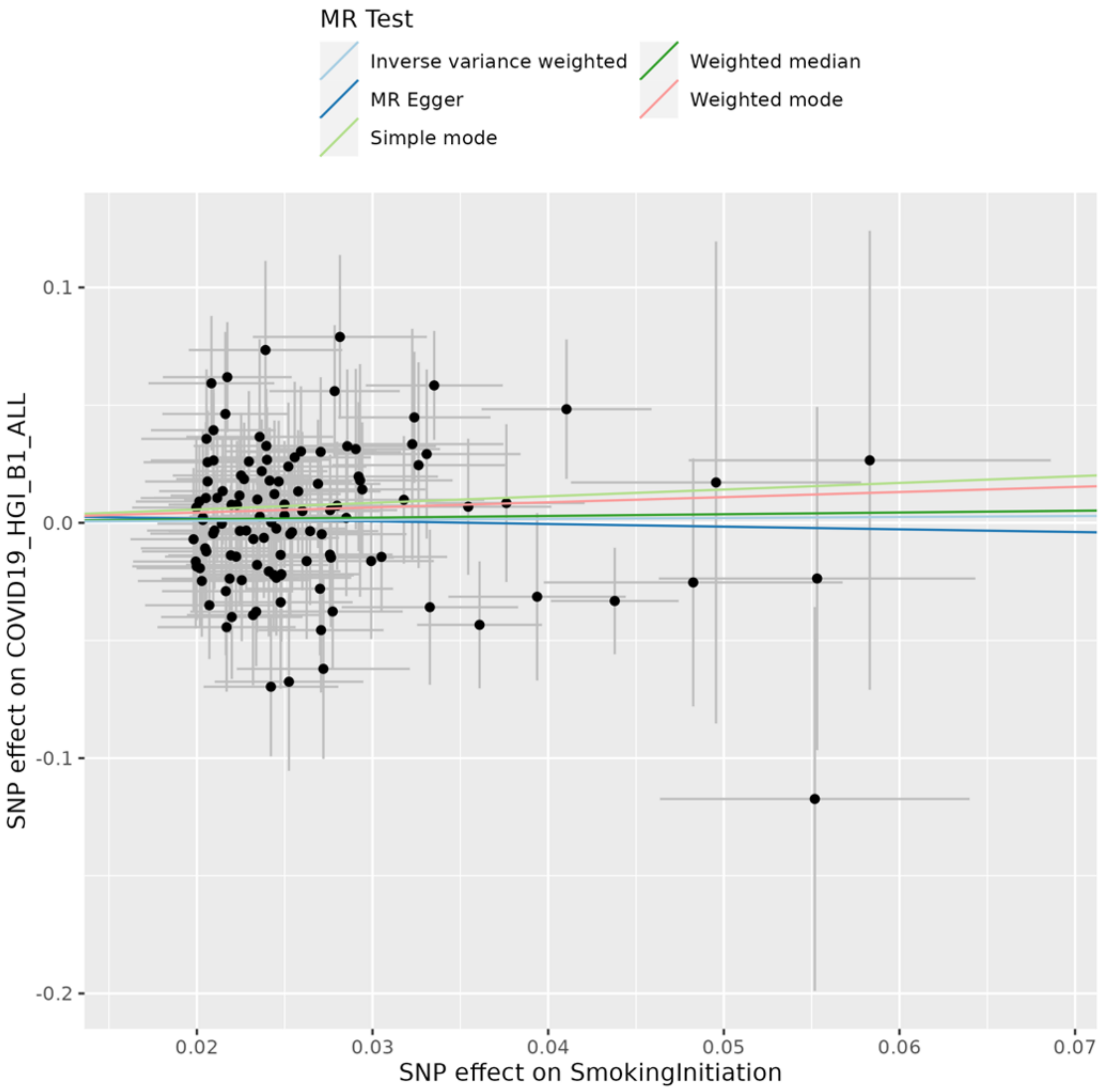

Fig R3.2 Scatter plot demonstrating the SNP effect of cigarettes smoked per day on COVID-19 severity (B1 ALL). Each dot represents one of 82 SNPs, with the SNP effect on cigarettes smoked per day plotted on the x-axis and the SNP effect on COVID-19 severity depicted on the y-axis. The vertical and horizontal lines encompassing each dot correspond to the confidence intervals for the SNP effects. Mendelian Randomization (MR) estimates from different methods are illustrated as solid lines across the scatter plot: light blue for the Inverse Variance Weighted method, green for the Weighted Median method, blue for the MR Egger method, red for the Weighted Mode method, and light green for the Simple Mode method. The intersection of these lines provides an overall estimate of the causal effect of cigarettes smoked per day on COVID-19 severity.

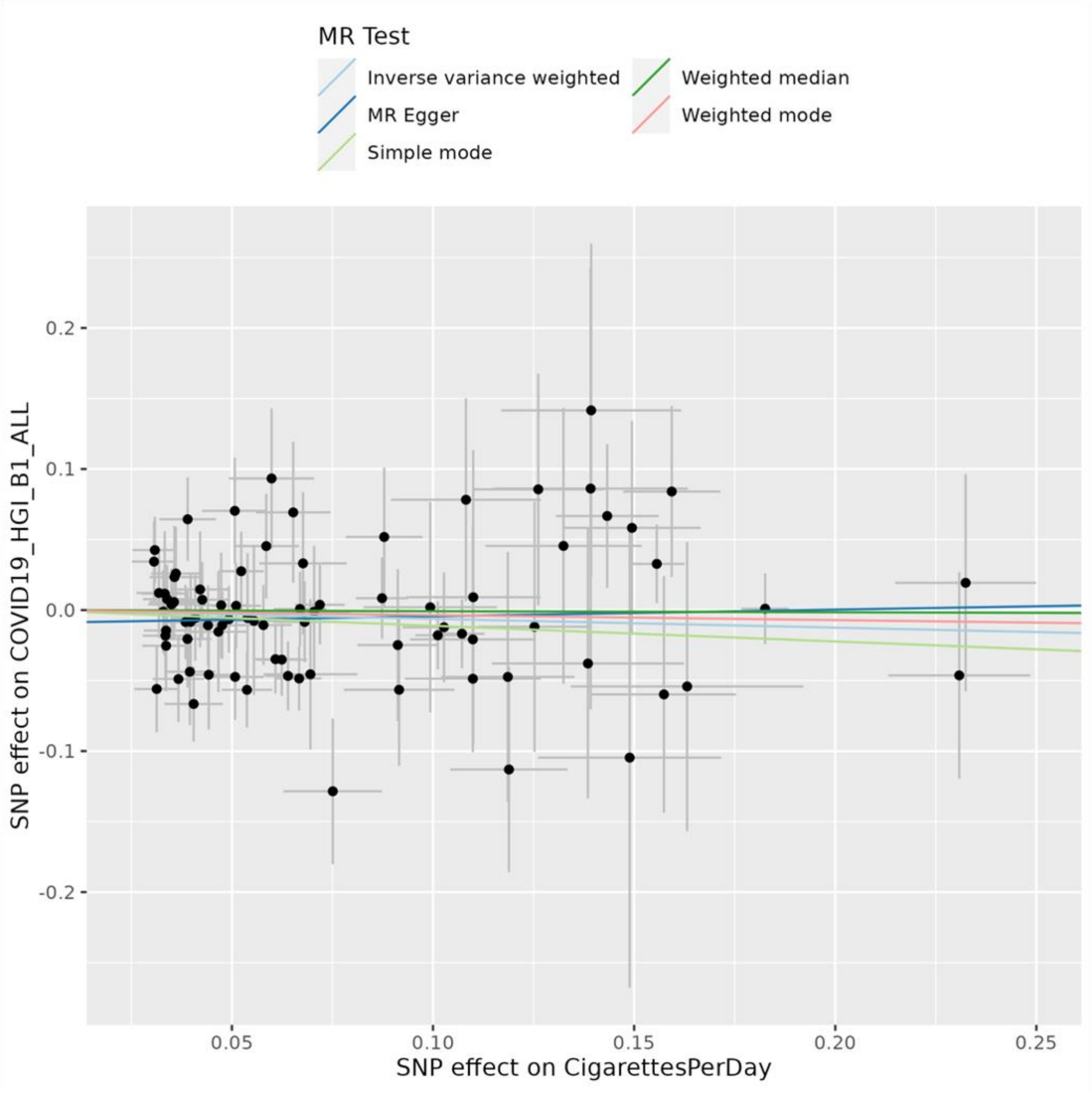

Fig R3.3 Scatter plot demonstrating the SNP effect of smoking initiation on COVID-19 susceptibility (B2 ALL). Each dot represents one of 116 SNPs, with the SNP effect on smoking initiation plotted on the x-axis and the SNP effect on COVID-19 severity depicted on the y-axis. The vertical and horizontal lines encompassing each dot correspond to the confidence intervals for the SNP effects. Mendelian Randomization (MR) estimates from different methods are illustrated as solid lines across the scatter plot: light blue for the Inverse Variance Weighted method, green for the Weighted Median method, blue for the MR Egger method, red for the Weighted Mode method, and light green for the Simple Mode method. The intersection of these lines provides an overall estimate of the causal effect of cigarettes smoked per day on COVID-19 severity.

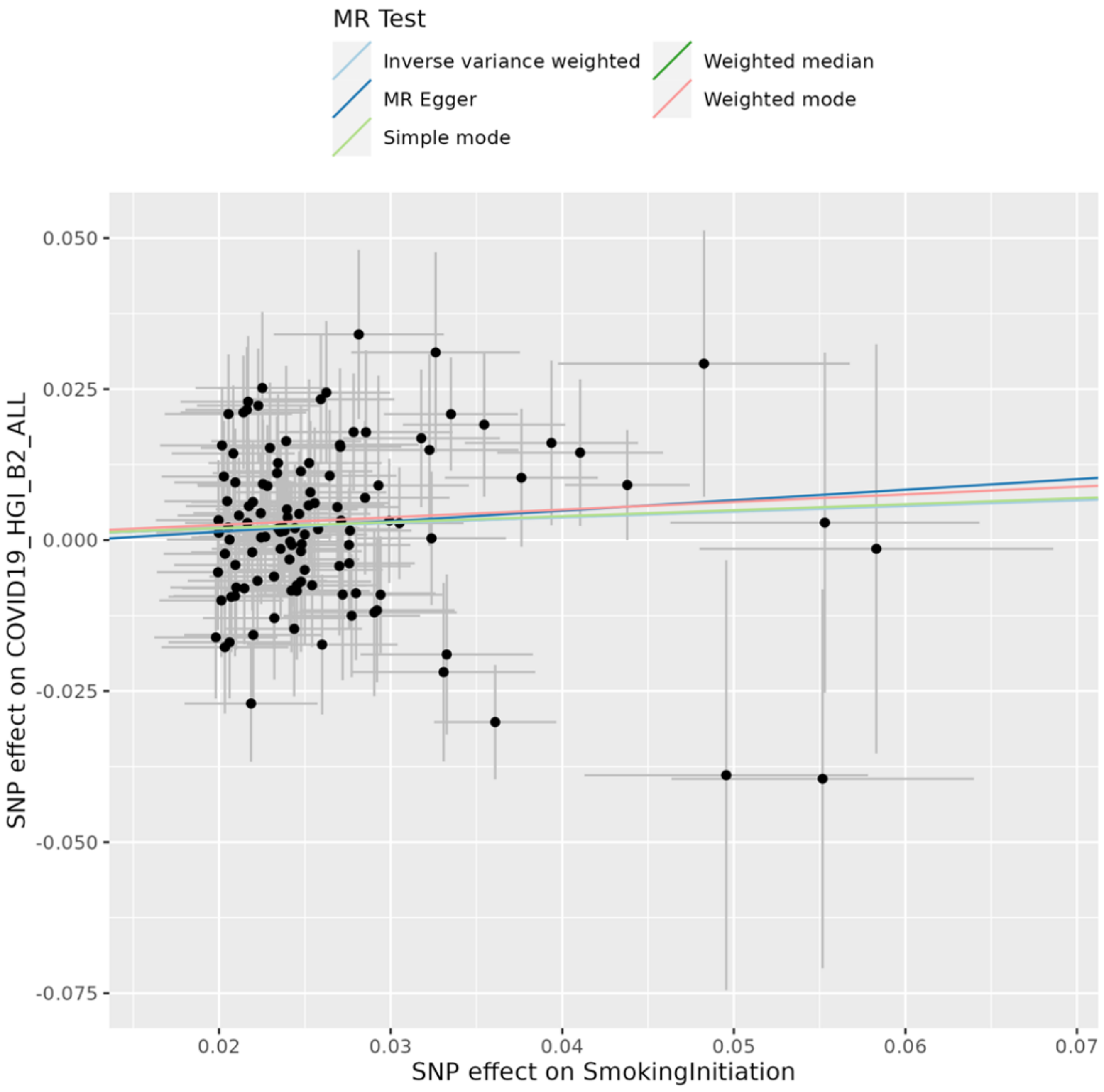

Fig R3.4 Scatter plot demonstrating the SNP effect of cigarettes smoked per day on COVID-19 susceptibility (B2 ALL). Each dot represents one of 83 SNPs, with the SNP effect on cigarettes smoked per day plotted on the x-axis and the SNP effect on COVID-19 severity depicted on the y-axis. The vertical and horizontal lines encompassing each dot correspond to the confidence intervals for the SNP effects. Mendelian Randomization (MR) estimates from different methods are illustrated as solid lines across the scatter plot: light blue for the Inverse Variance Weighted method, green for the Weighted Median method, blue for the MR Egger method, red for the Weighted Mode method, and light green for the Simple Mode method. The intersection of these lines provides an overall estimate of the causal effect of cigarettes smoked per day on COVID-19 severity.

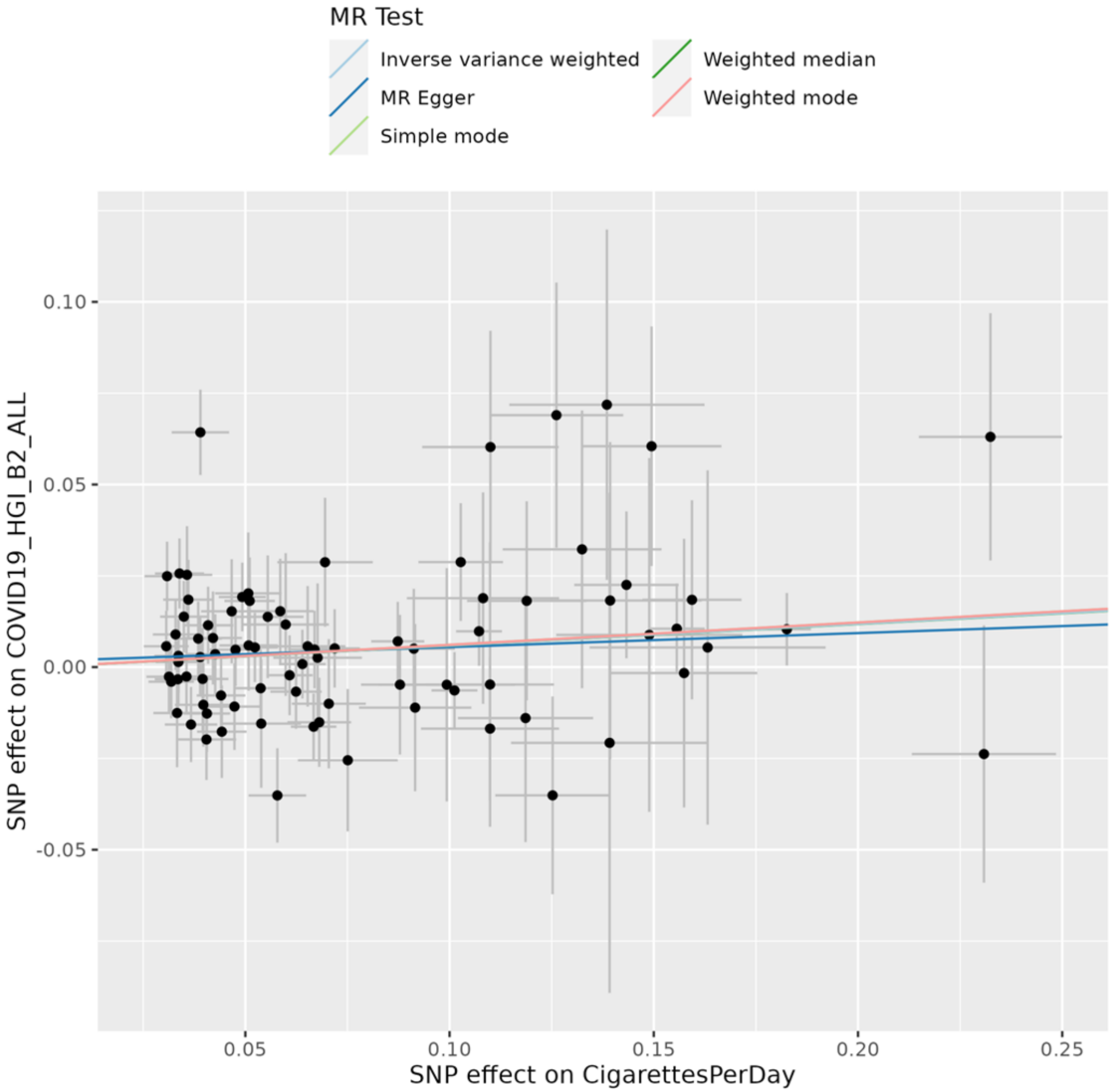

Table R3.1 Mendelian randomization analysis for the effect of smoking initiation on COVID-19 severity (117 SNPs). MR-PRESSO detected no outlier.

Method/Analysis	Beta	Standard Error	P-value
MR Egger	-0.11	0.51	0.82
Weighted median	0.07	0.15	0.63
Inverse variance weighted	0.04	0.10	0.69
Simple mode	0.28	0.43	0.51
Weighted mode	0.22	0.44	0.62
MR-PRESSO Raw	0.06	0.10	0.54

Table R3.2 Mendelian randomization analysis for the effect of cigarettes consumed per day on COVID-19 severity (82 SNPs). MR-PRESSO detected no outlier.

Method/Analysis	Beta	Standard Error	P-value
MR Egger	0.05	0.11	0.66
Weighted median	-0.01	0.09	0.93
Inverse variance weighted	-0.06	0.05	0.25
Simple mode	-0.11	0.18	0.53
Weighted mode	-0.04	0.10	0.73
MR-PRESSO Raw	-0.07	0.05	0.20

Table R3.3 Mendelian randomization analysis for the effect of smoking initiation on COVID-19 susceptibility (116 SNPs). MR-PRESSO detected no outlier.

Method/Analysis	Beta	Standard Error	P-value
MR Egger	0.17	0.22	0.43
Weighted median	0.09	0.06	0.13
Inverse variance weighted	0.09	0.04	0.03
Simple mode	0.10	0.16	0.54
Weighted mode	0.13	0.14	0.37
MR-PRESSO Raw	0.08	0.04	0.05

Table R3.4 Mendelian randomization analysis for the effect of cigarettes consumed per day on COVID-19 susceptibility (83 SNPs). MR-PRESSO detected one outlier.

Method/Analysis	Beta	Standard Error	P-value
MR Egger	0.04	0.05	0.46
Weighted median	0.06	0.03	0.09
Inverse variance weighted	0.06	0.03	0.02
Simple mode	0.06	0.07	0.36
Weighted mode	0.06	0.04	0.11
MR-PRESSO Raw	0.06	0.03	0.03
MR-PRESSO Outlier-corrected	0.05	0.02	0.03

3.2. PRS Generation: The study exclusively utilized PRS-CS for generating PRS, overlooking the PRS-CSX method which is known to be more suitable for multiple ancestries. This choice might limit the generalizability of the findings across diverse populations.

We thank the reviewer for their input on our choice of PRS generation method. We chose PRS-CS over PRS-CSX due to the homogeneous European ancestry of our cohorts, where the benefits of PRS-CSx are less pronounced. The absence of diverse GWAS data on COVID-19 severity and susceptibility limits the utility of PRS-CSx in this context. We have added to the discussion (pp 25 – 26) the limitation of our approach and the need for inclusive genetic research to improve the generalizability of PRS findings across ancestries (see response R1.2):

"This underscores the need to establish larger, more diverse populations, particularly by including more representative non-European samples and applying ancestry-aware PRS methods, thereby enhancing the accuracy and broader applicability of COVID-19 severity PRS investigations across diverse ethnic groups."

3.3. COVID Severity PRS Link with Tobacco Use Disorder: The manuscript highlighted a significant association between COVID-19 severity PRS and tobacco use disorder in both B1_ALL and B2_ALL analyses. This finding raises questions regarding the causal relationship between the two, warranting further investigation through MR to understand the underlying causality.

Given the insights from our MR analyses, we have expanded our discussion to address the association between COVID-19 severity PRS and tobacco use disorder. Our MR findings consistently showed no significant causal link between smoking initiation and COVID-19 severity. For COVID-19 susceptibility, the evidence of a potential causal link with the number of cigarettes smoked per day is weak and inconsistent across MR methods. The borderline significant results from the Inverse Variance Weighted method and MR-PRESSO are acknowledged; however, they do not provide compelling evidence for a strong causal relationship. This nuanced interpretation of the results underscores the complexity of the relationship between tobacco use and COVID-19 outcomes, which we discuss in our response to comment R3.1 above.

3.4. The study presents an approach to understanding the genetic predispositions to COVID-19 severity, offering valuable insights into managing risks associated with the disease. However, it is imperative to address the limitations and to substantiate the findings through causal inference studies to enhance the robustness and applicability of the approach in real-world settings.

We concur with the importance of acknowledging the study's limitations and the need for substantiating findings through causal inference studies. The MR analyses added to the supplementary materials serve to address this concern partially. While the MR approach strengthens the causal inferences that can be drawn from our study, we recognize that these findings are inconclusive and require follow-up with well-powered studies.

We added the following to our paragraph on the MR analysis in the Discussion (p 22):
"While the MR approach strengthens the causal inferences that can be drawn from our study, we recognize that these findings are inconclusive and require follow-up with well-powered studies to understand the implications of our results fully."
